# Anti-inflammatory and antioxidant effects of dipotassium glycyrrhizinate in acute respiratory distress syndrome

**DOI:** 10.3389/fmed.2026.1690322

**Published:** 2026-03-04

**Authors:** Wei Cao, Dongjun Xu, Huijie Yu, Xuning Shen

**Affiliations:** Emergency Medicine Department, Affiliated Hospital of Jiaxing University, Jiaxing University, Jiaxing, Zhejiang, China

**Keywords:** animal experiment, anti-inflammatory, antioxidant, ARDS, dipotassium glycyrrhizinate

## Abstract

**Introduction:**

Acute respiratory distress syndrome (ARDS) is a severe clinical syndrome driven by inflammation, oxidative stress, and pulmonary tissue injury, for which effective therapy drugs remain lacking. In this study, the therapeutic potential and underlying mechanisms of dipotassium glycyrrhizinate (DG) in ARDS were systematically evaluated through both *in vitro* and *in vivo* experiments.

**Methods and results:**

In an A549 cell model, DG exhibited no cytotoxicity within the tested concentration range and significantly suppressed LPS-induced excessive reactive oxygen species (ROS) generation and pro-inflammatory cytokine expression, including Tumor necrosis factor (TNF)-*α*,and Interleukin (IL)-6, while upregulating the anti-inflammatory cytokine IL-10, indicating its potent anti-inflammatory and antioxidant properties. In an LPS-induced ARDS mouse model, DG treatment not only significantly reduced serum levels of inflammatory cytokines but also increased the activity of the antioxidant enzyme superoxide dismutase (SOD), decreased the levels of myeloperoxidase (MPO) and malondialdehyde (MDA), and markedly alleviated pulmonary histopathological damage, demonstrating notable tissue-protective effects. Based on these findings, network pharmacology analysis revealed that DG targeted multiple ARDS-related core proteins (EGFR, MAPK1, FGFR1) enriched in key signaling pathways such as PI3K-AKT, EGFR, and HIF-1. Molecular docking and molecular dynamics simulations further confirmed the stable binding and strong affinity between DG and EGFR, supporting a regulatory mechanism in the context of ARDS pathogenesis.

**Discussion:**

In conclusion, DG alleviates ARDS-associated inflammation and oxidative stress through coordinated modulation of multiple signaling pathways, providing a theoretical and experimental foundation for its potential development as a natural therapeutic agent against ARDS.

## Introduction

Acute respiratory distress syndrome (ARDS) is a severe clinical condition caused by infections, trauma, inhalation injuries, or sepsis, characterized by acute onset, bilateral non-cardiogenic pulmonary infiltrates, and a PaO₂/FiO₂ ratio < 300 mmHg under PEEP ≥ 5 cm H₂O (1, 2). According to the Berlin definition, ARDS is classified as mild (201–300 mmHg), moderate (101–200 mmHg), or severe (≤ 100 mmHg) (3). Its complex pathogenesis involves alveolar–capillary barrier disruption, massive release of inflammatory mediators (e.g., TNF-*α*, IL-1β, IL-6), neutrophil infiltration, oxidative stress, and apoptosis, leading to reduced lung compliance, alveolar collapse, and impaired gas exchange (4). Pathological findings often reveal diffuse alveolar damage (DAD), with mortality reaching 71.9% in patients with DAD compared to 45.5% in those without. Despite advances in supportive care—such as low tidal volume ventilation, fluid management, PEEP optimization, and prone positioning—no pharmacological therapy has been widely recognized in the past decade. The LUNG SAFE study, covering 459 ICUs across 50 countries, reported that 10.4% of ICU patients met ARDS criteria, rising to 23.4% among those receiving mechanical ventilation. Hospital mortality ranged from 34.9% (mild) to 46.1% (severe), with a weighted average of 39.4% (5). A 2024 analysis further reported 30-day mortality rates of 10.5, 11.6, and 18.1% for mild, moderate, and severe ARDS, respectively, indicating a strong correlation between severity and prognosis (6). In summary, ARDS remains a major challenge in critical care due to its high incidence, high mortality, and frequent long-term sequelae—such as impaired diffusion capacity in ~80% of survivors—and the absence of effective targeted pharmacotherapies.

Dipotassium glycyrrhizinate (DG) is a water-soluble salt of glycyrrhizic acid, a major triterpenoid saponin derived from the roots and rhizomes of *Glycyrrhiza uralensis Fisch*. Glycyrrhizic acid is typically obtained by aqueous or hydroalcoholic extraction, followed by purification using adsorption or chromatographic methods. Subsequent alkaline hydrolysis and neutralization with potassium ions produce DG, which shows improved water solubility and bioavailability (7). Owing to these physicochemical advantages, DG has been widely used in pharmaceutical formulations and has received increasing attention for its broad pharmacological activities (8). DG has demonstrated anti-inflammatory, antioxidant, immunomodulatory, and cytoprotective effects, which are supported by numerous *in vitro* and *in vivo* studies (9). For example, in RAW 264.7 macrophages, DG significantly inhibits the release of HMGB1 and suppresses the expression of pro-inflammatory cytokines such as TNF-*α*, IL-1β, and IL-6, along with the inactivation of NF-κB and MAPK signaling pathways—effectively reducing inflammatory damage (10). In murine models of colitis, DG markedly alleviates histological inflammation and mucosal injury, and downregulates HMGB1, its receptors (e.g., TLR4, RAGE), and related cytokine mRNA levels (11, 12). Furthermore, in rat models of cutaneous wound healing, topical administration of DG significantly accelerates re-epithelialization and collagen deposition while decreasing mRNA expression of COX-2, TNF-*α*, IL-1α, IL-6, IL-8, and NF-κB, and upregulating IL-10 during the treatment period (days 3, 7, and 14), indicating potent anti-inflammatory and tissue-regenerative properties (13). Additional reviews and molecular docking studies have also reported that *Glycyrrhizin* derivatives (e.g., Ammonium Glycyrrhizinate) exert sustained anti-inflammatory effects through inhibition of COX-2, mPGEs, or NF-κB signaling, significantly reducing paw edema and hyperalgesia in zymosan-induced models (14). However, these investigations have largely focused on localized or chronic inflammatory conditions outside the pulmonary context. To date, the therapeutic potential and mechanistic role of DG in acute lung injury (ALI) or ARDS, which are characterized by overwhelming inflammation, oxidative stress, and alveolar barrier disruption, have not been systematically explored. Therefore, the present study addresses this critical knowledge gap by evaluating DG in ARDS-relevant cellular and animal models and elucidating its regulatory effects on key inflammatory and injury in the lung.

Based on the above background, this study aims to systematically evaluate the therapeutic effects of DG in both *in vitro* cell models and *in vivo* ARDS animal models, with a particular focus on its regulation of pulmonary inflammation, oxidative stress markers, and histopathological injury. Furthermore, integrated computational approaches—including network pharmacology and molecular dynamics simulations—will be employed to predict and identify potential targets and key signaling pathways. Through a combined *in vitro* and *in vivo* research strategy, this study seeks to clarify the mechanistic pathways and pharmacological effects of DG in modulating ARDS-related pathological processes. These efforts will lay a solid foundation for modern pharmacological understanding and provide reproducible experimental evidence and methodological support for future investigations into dose–response relationships, optimal timing of administration, and combination treatment strategies. Ultimately, this study aims to support the development of DG as a potential targeted therapeutic agent for ARDS by offering rigorous theoretical and experimental validation.

## Results

### DG attenuated LPS-induced oxidative stress and inflammatory responses in A549 cells

**Figure 1:**
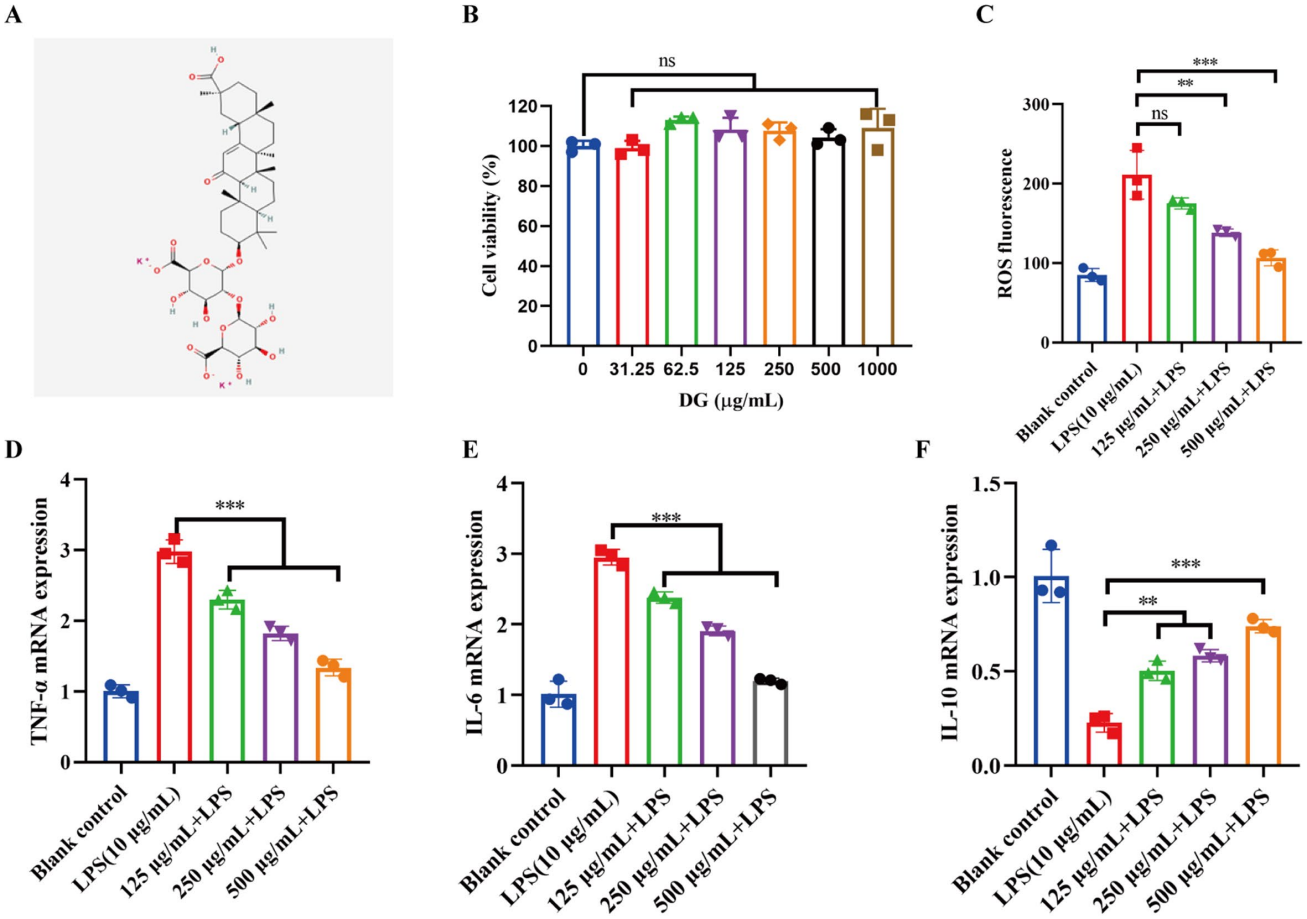
Effects of dipotassium glycyrrhizinate (DG) on LPS-induced oxidative stress and inflammatory responses in A549 cells. **(A)** Chemical structure of DG. **(B)** Cell viability of A549 cells after 24 h treatment with varying concentrations of DG (0–1,000 μg/mL), assessed by the CCK-8 assay. **(C)** Intracellular reactive oxygen species (ROS) levels detected using the DCFH-DA fluorescent probe in control and LPS-treated groups, with or without DG pretreatment. **(D–F)** Relative mRNA expression levels of TNF-*α* **(D)**, IL-6 **(E)**, and IL-10 **(F)** in each group, as determined by quantitative real-time PCR (qRT-PCR). Data are presented as mean ± standard deviation (*n* = 3). Statistical significance was determined using one-way ANOVA; ns, not significant; **p < 0.01, ***p < 0.001.

To investigate the potential protective effects of DG in ARDS, we first performed preliminary evaluations using an LPS-induced A549 cell model. As illustrated in Figure 1A, DG is a water-soluble derivative formed by the binding of two potassium ions to the triterpenoid saponin backbone of glycyrrhizic acid. The presence of multiple carboxyl and hydroxyl groups endows DG with high hydrophilicity (15). This hydrophilic character, combined with its potassium salt form, significantly enhances aqueous solubility, facilitating rapid dissolution and formulation. Additionally, the salt form improves physicochemical stability in biological fluids, minimizing precipitation and potentially increasing absorption rate and bioavailability upon oral or parenteral administration (16). Given that potassium is a major physiological cation, its salt form often exhibits favorable systemic distribution, suggesting that DG may possess balanced tissue distribution properties *in vivo*, which is advantageous for treating systemic inflammation or acute lung injury. Cell viability assays showed that DG, within the concentration range of 31.25–1,000 μg/mL, exhibited no significant cytotoxicity to A549 cells compared to the untreated control, thereby defining a safe concentration window for subsequent functional and mechanistic investigations (Figure 1B). Preliminary experiments showed that DG alone, across the tested concentration range, did not significantly affect cell viability; therefore, subsequent experiments focused on evaluating its effects under LPS stimulation. In the assessment of oxidative stress, LPS stimulation significantly elevated intracellular reactive oxygen species (ROS) levels, whereas DG treatment at 125, 250, and 500 μg/mL markedly reduced ROS fluorescence intensity in a dose-dependent manner (Figure 1C). At the transcriptional level, LPS markedly upregulated pro-inflammatory cytokines TNF-*α* and IL-6, while DG treatment significantly suppressed their expression in a concentration-dependent fashion (Figures 1D–F). Concurrently, LPS stimulation led to a pronounced reduction in the expression of the anti-inflammatory cytokine IL-10, which was significantly reversed by DG treatment across all tested concentrations. Collectively, these data demonstrated that DG, at non-cytotoxic concentrations, effectively attenuated LPS-induced oxidative stress and pro-inflammatory cytokine upregulation, while restoring anti-inflammatory IL-10 expression in alveolar epithelial cells, indicating its potent anti-inflammatory and antioxidant protective effects *in vitro*.

### DG attenuated systemic inflammation and ameliorates lung histopathological injury in LPS-induced ARDS mice

**Figure 2:**
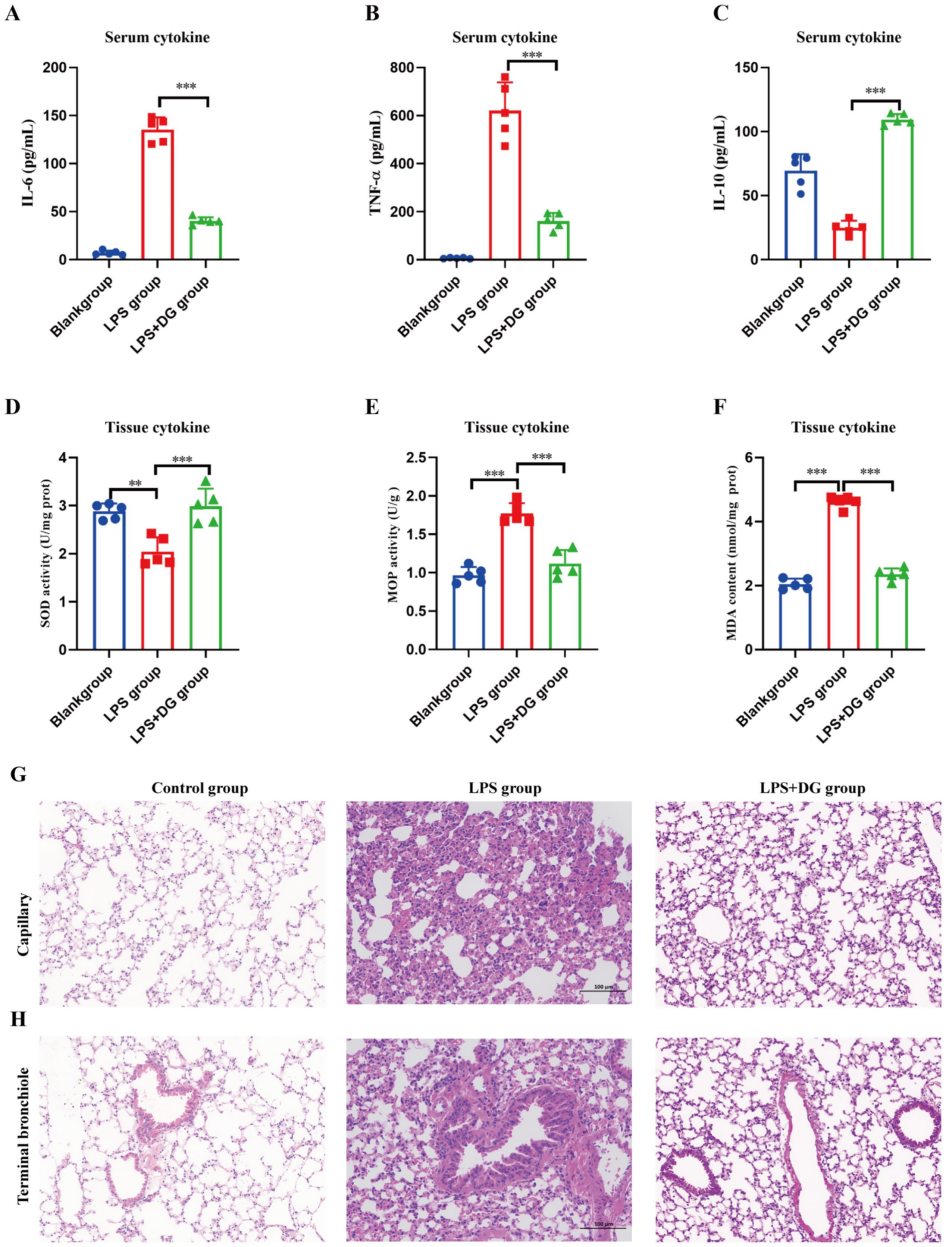
Therapeutic effects of DG in an LPS-induced ARDS mouse model. **(A–C)** Serum levels of IL-6 **(A)**, TNF-α **(B)**, and IL-10 **(C)** were measured by ELISA. **(D–F)** Oxidative stress markers in lung tissues were assessed, including superoxide dismutase (SOD) activity **(D)**, myeloperoxidase (MPO) activity **(E)**, and malondialdehyde (MDA) content **(F)**. **(G,H)** Representative hematoxylin and eosin (H&E) staining images of mouse lung tissues showing the capillary region **(G)** and terminal bronchiole area **(H)** in different groups (scale bar = 100 μm). Data are presented as mean ± SD (*n* = 5). Statistical analysis was performed using one-way ANOVA; **p < 0.01, ***p < 0.001.

To further evaluate the therapeutic potential of DG *in vivo*, a mouse model of acute lung injury induced by LPS was established. Systemic inflammation, oxidative stress in lung tissues, and histopathological changes were comprehensively assessed. As shown in Figures 2A–C, serum levels of IL-6, TNF-*α*, and IL-10 remained at low baseline levels in the control group. Upon LPS treatment, the pro-inflammatory cytokines IL-6 (Figure 2A) and TNF-α (Figure 2B) were significantly elevated, reflecting a robust systemic inflammatory response. A DG-only group was not included in the animal experiments due to animal welfare considerations. However, DG treatment markedly reduced the levels of both cytokines, indicating its potent anti-inflammatory effect. For the anti-inflammatory cytokine IL-10 (Figure 2C), LPS stimulation led to a significant decrease compared to the control, whereas DG administration significantly restored IL-10 levels, suggesting a favorable role in promoting anti-inflammatory balance. Oxidative stress markers in lung tissues further illustrated the antioxidant potential of DG (Figures 2D–F). LPS significantly reduced the activity of superoxide dismutase (SOD; Figure 2D), while DG administration restored SOD activity to near-normal levels, indicating enhanced ROS scavenging capacity. Myeloperoxidase (MPO) activity (Figure 2E), indicative of neutrophil infiltration, was markedly increased after LPS exposure, but significantly attenuated by DG treatment. Similarly, malondialdehyde (MDA) content (Figure 2F), a marker of lipid peroxidation, was elevated in the LPS group and significantly decreased following DG intervention, suggesting reduced oxidative damage to cellular membranes. Histological evaluation (Figures 2G,H) provided direct evidence of DG’s protective effect on lung architecture. Hematoxylin and eosin (H&E) staining showed that the control group maintained normal alveolar structure with thin septa and clear airspaces (Figures 2G,H). In contrast, LPS administration caused typical features of acute lung injury, including thickened alveolar septa, capillary congestion, extensive inflammatory cell infiltration, alveolar collapse, and disorganized architecture (Figures 2G,H). Remarkably, DG treatment ameliorated these pathological alterations, evidenced by relatively preserved alveolar structure, reduced septal thickening, diminished inflammatory infiltration, and restored airspace integrity (Figures 2G,H). Collectively, DG significantly inhibited pro-inflammatory cytokine release, restored anti-inflammatory IL-10 levels, reduced oxidative stress markers, enhanced antioxidant enzyme activity, and alleviated lung histopathological damage in LPS-induced ARDS mice. These *in vivo* findings are highly consistent with the *in vitro* results and provide solid experimental support for DG as a promising therapeutic candidate for ARDS.

### Network pharmacology analysis reveals multi-target and multi-pathway mechanisms of DG in ARDS

**Figure 3:**
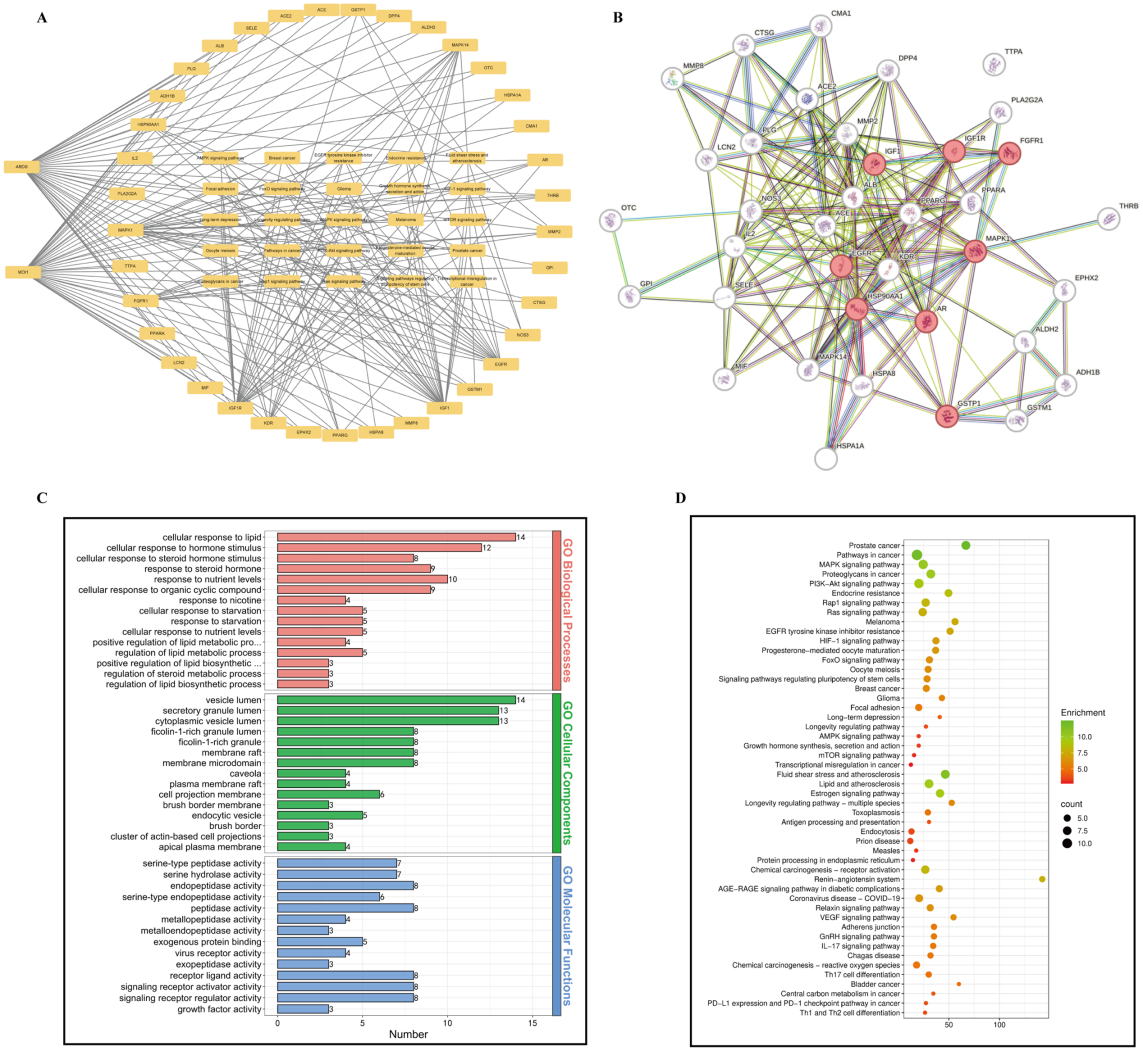
Network pharmacology analysis reveals the potential mechanisms of DG in ARDS. **(A)** Disease–target–pathway interaction network. Circles represent the shared targets of DG and ARDS, with signaling pathways in the middle and connecting lines indicating target–pathway associations. **(B)** Protein–protein interaction (PPI) network. Red nodes indicate the eight hub targets (MAPK1, EGFR, IGF1R, FGFR1, PPARG, HSP90AA1, GSTP1, and MAPK14), and node size reflects degree centrality. **(C)** Gene ontology (GO) enrichment analysis, including the top 30 significantly enriched terms in biological process (BP), cellular component (CC), and molecular function (MF) categories. **(D)** Kyoto encyclopedia of genes and genomes (KEGG) pathway enrichment analysis. Bubble color represents enrichment significance (enrichment value), and bubble size corresponds to the number of involved targets (count).

To systematically elucidate the potential therapeutic mechanisms of DG in ARDS, we employed a network pharmacology approach to construct a comprehensive “disease–target–pathway” multilayer interaction network based on intersecting genes. In this network, the peripheral nodes represented common targets shared between DG and ARDS, the central region illustrated enriched signaling pathways, and the connecting lines depicted the interactions between targets and pathways (Figure 3A). Key targets included MAPK1, AKT1, EGFR, TNF, IL2, FGFR1, MMP2, and PPARA, which were involved in critical pathological processes such as inflammation regulation, oxidative stress, apoptosis, and tissue repair (Figure 3A). These targets were associated with several classical pathways, including MAPK, PI3K-AKT, TNF, mTOR, HIF-1, and EGFR signaling, as well as pathways related to metabolism, immune regulation, and cell cycle control, such as FoxO, endocrine resistance, and EGFR tyrosine kinase inhibitor resistance (Figure 3A). Notably, the EGFR signaling pathway was centrally positioned in the network, linking key nodes including EGFR, MAPK1, and AKT1, suggesting its potential involvement in promoting alveolar epithelial repair and restoring barrier function. To further identify key regulatory hubs, a protein–protein interaction (PPI) network was constructed. Analysis revealed eight hub genes with high connectivity (MAPK1, EGFR, IGF1R, FGFR1, PPARG, HSP90AA1, GSTP1, and MAPK14), forming tightly clustered modules associated with inflammation, immune modulation, and tissue repair, indicating their central roles in coordinating multi-pathway regulatory processes (Figure 3B). Gene Ontology (GO) enrichment analysis was subsequently performed to explore the biological significance of these shared targets (Figure 3C). In the biological process (BP) category, genes were enriched in lipid response (14 genes), response to hormone stimulus (12 genes), response to steroid hormones (10 genes), and regulation of lipid metabolism (Figure 3C). In the cellular component (CC) category, target genes were predominantly localized to vesicle lumen (14 genes), secretory granule lumen (13 genes), plasma membrane microdomains, and membrane rafts (Figure 3C). In the molecular function (MF) category, enrichment was observed in serine-type endopeptidase activity (7 genes), hydrolase activity, receptor–ligand activity, and growth factor activity (Figure 3C). These findings implied that DG may exert therapeutic effects in ARDS by regulating lipid/hormone responses, vesicle- and membrane-related signal transduction, protease activity, and growth factor signaling. Subsequent KEGG pathway enrichment analysis further identified key signaling networks potentially modulated by DG. These include MAPK, PI3K-AKT, EGFR, HIF-1, mTOR, Ras, and Rap1 signaling pathways, all of which are crucial in mediating inflammation, apoptosis, oxidative stress, and tissue repair (Figure 3D). In addition, the targets were significantly enriched in immune-related pathways (e.g., antigen processing and presentation, Th17 cell differentiation, IL-17 signaling, and coronavirus disease–COVID-19), as well as metabolic and vascular function pathways (e.g., AGE–RAGE signaling, lipid and atherosclerosis) (Figure 3D). Collectively, these results suggested that DG may exert pleiotropic effects in ARDS by attenuating inflammatory and oxidative stress responses while activating pathways associated with epithelial repair and barrier restoration, thereby contributing to a comprehensive multi-target, multi-pathway therapeutic mechanism.

### Molecular docking analysis of DG with core ARDS targets

**Figure 4:**
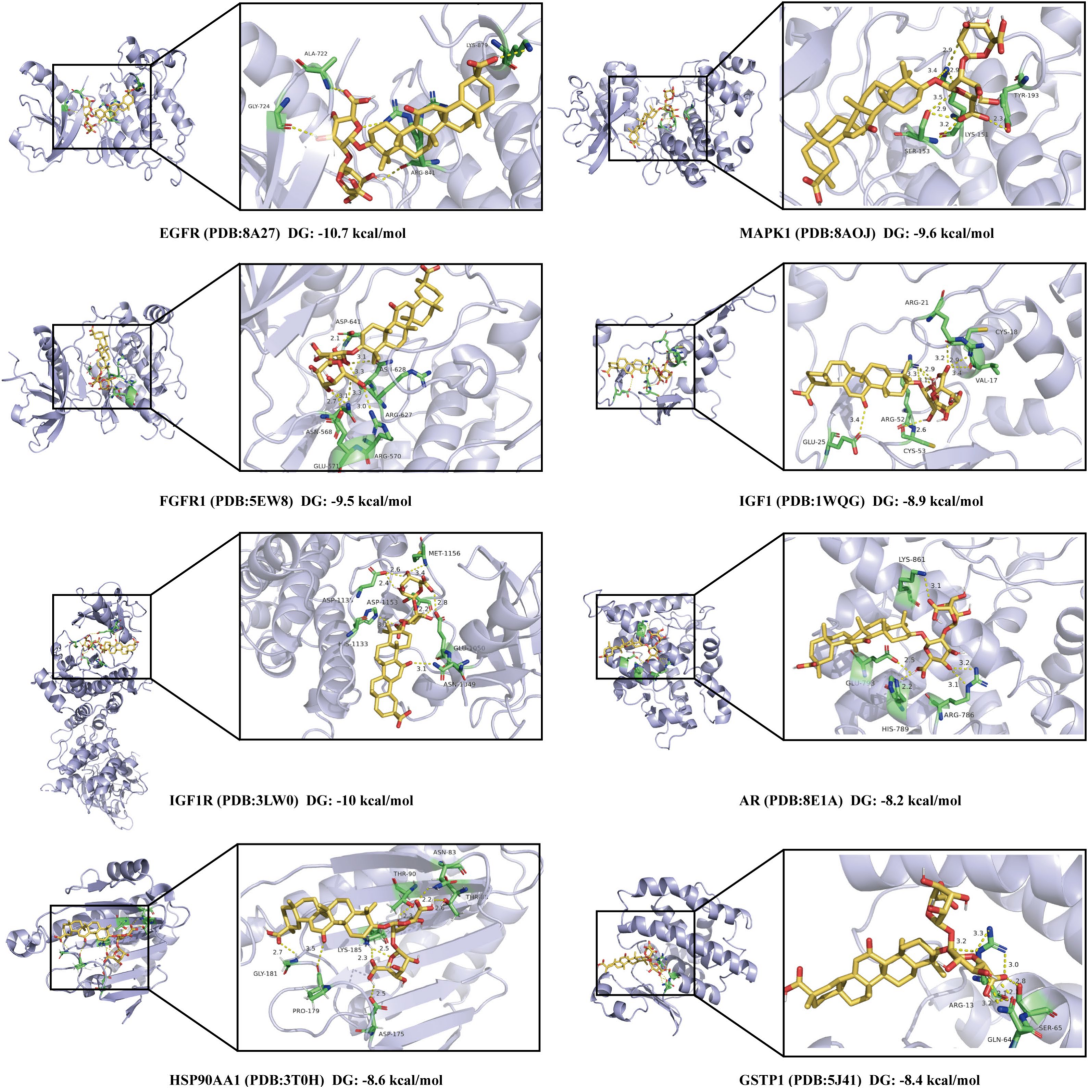
Molecular docking analysis of DG with eight core targets associated with ARDS. The core targets include EGFR (PDB: 8A27, −10.7 kcal/mol), MAPK1 (PDB: 8AOJ, −9.6 kcal/mol), FGFR1 (PDB: 5EW8, −9.5 kcal/mol), IGF1 (PDB: 1WQG, −8.9 kcal/mol), IGF1R (PDB: 3LWO, −10.0 kcal/mol), AR (PDB: 8E1A, −8.2 kcal/mol), HSP90AA1 (PDB: 3T0H, −8.6 kcal/mol), and GSTP1 (PDB: 5H4I, −8.4 kcal/mol). Left panels show the overall binding conformations of DG within the active sites of each protein, while the right panels display magnified views highlighting key amino acid residues involved in hydrogen bonding and other intermolecular interactions.

Based on the results of network pharmacology, eight core regulatory targets associated with ARDS were identified, including MAPK1, EGFR, IGF1R, FGFR1, PPARG, HSP90AA1, GSTP1, and MAPK14. To verify the binding potential and interaction modes between DG and these targets, molecular docking simulations were conducted to assess the affinity and structural compatibility of DG with the active pockets of each protein. As shown in Figure 4, all binding energies between DG and the core targets were negative, indicating spontaneous and favorable binding. Notably, DG exhibited the strongest affinity toward EGFR (PDB: 8A27) with a binding energy of −10.7 kcal/mol, followed by IGF1R (PDB: 3LWO, −10.0 kcal/mol), FGFR1 (PDB: 5EW8, −9.5 kcal/mol), and MAPK1 (−9.6 kcal/mol). Other targets, including IGF1 (−8.9 kcal/mol), HSP90AA1 (−8.6 kcal/mol), GSTP1 (−8.4 kcal/mol), and AR (−8.2 kcal/mol), also demonstrated binding energies below −8.0 kcal/mol, suggesting high-affinity interactions (Figure 4). Binding site analyses revealed that DG forms multiple stabilizing interactions within the active sites of these proteins, including hydrogen bonds, hydrophobic contacts, and *π*–π stacking (Figure 4). Specifically, in the EGFR active pocket, DG established multiple hydrogen bonds with key residues such as ALA722, GLA724, and LYS879, and also engaged in hydrophobic interactions with residues like LEU844 and VAL726, thereby stabilizing the overall binding conformation (Figure 4). Similar multivalent interactions were observed in the binding modes with MAPK1, IGF1R, and FGFR1, supporting the capacity of DG to modulate their activity through stable binding (Figure 4). In summary, molecular docking results confirmed that DG possesses strong and stable binding potential to multiple ARDS-related targets, supporting its polypharmacological profile predicted by network pharmacology. Together, these results support a mechanism by which DG may exert therapeutic effects in ARDS through coordinated multi-target and multi-pathway regulation.

### Molecular dynamics simulation and MM-PBSA binding energy decomposition analysis of DG with EGFR

**Figure 5:**
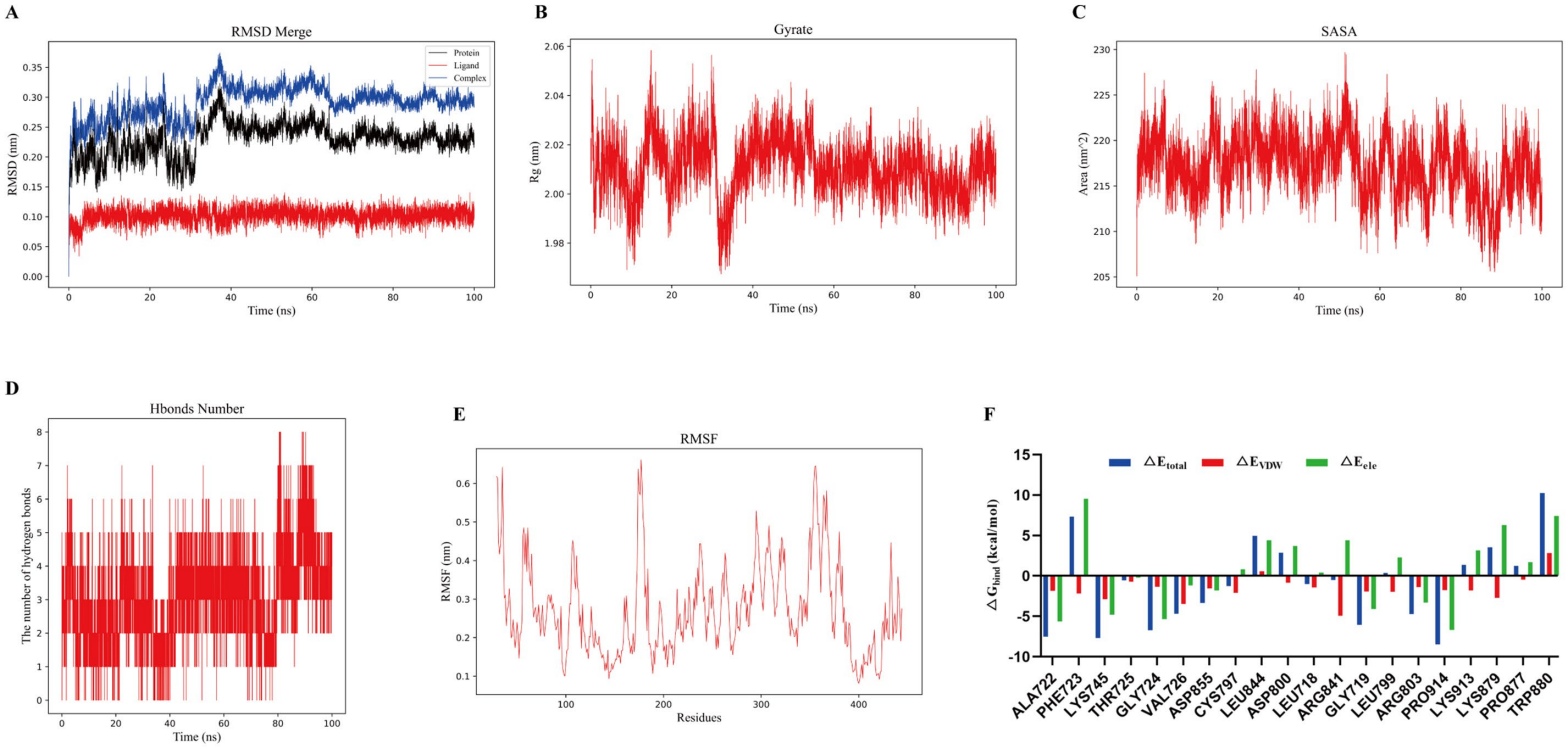
Molecular dynamics simulation reveals the binding stability and energy decomposition of DG with EGFR. **(A)** Root-mean-square deviation (RMSD) trajectories of the protein, ligand, and complex over 100 ns, indicating overall structural stability. **(B)** Radius of gyration (Rg) of the complex during the simulation, reflecting global compactness of the protein structure. **(C)** Solvent-accessible surface area (SASA) trajectory of the complex, evaluating changes in surface exposure. **(D)** Number of hydrogen bonds between DG and EGFR over time, indicating the stability of intermolecular interactions. **(E)** Root-mean-square fluctuation (RMSF) analysis of EGFR residues, reflecting local flexibility and the stabilizing effect of ligand binding. **(F)** MM-PBSA binding free energy decomposition analysis of key residues at the EGFR binding interface. ΔE_total_, Total binding free energy; ΔE_VDW_, van der Waals interaction energy; ΔE_ele_, Electrostatic interaction energy.

Among the eight core targets identified, DG exhibited the strongest binding affinity to EGFR, with the lowest docking energy (−10.7 kcal/mol), suggesting a highly favorable interaction. To further evaluate the stability and interaction mechanism of the DG–EGFR complex, a 100 ns molecular dynamics (MD) simulation was performed, followed by structural and energetic analyses. The RMSD trajectories indicated that the protein, ligand, and complex rapidly reached equilibrium and remained within a narrow fluctuation range throughout the simulation (complex RMSD stabilized around 0.25–0.30 nm), reflecting the structural stability of the DG–EGFR binding (Figure 5A). The radius of gyration (Rg) fluctuated around 2.0 nm, suggesting that the overall protein conformation remained compact (Figure 5B). Solvent-accessible surface area (SASA) analysis showed that the surface exposure remained stable at approximately 200–230 nm^2^, indicating that ligand binding did not induce significant changes in protein solvent exposure (Figure 5C). Hydrogen bond analysis revealed that DG maintained 2–6 hydrogen bonds with EGFR consistently during the simulation, indicating stable and persistent intermolecular interactions (Figure 5D). RMSF analysis showed that most residues exhibited low fluctuation amplitudes, with higher flexibility limited to loop regions (Figure 5E). Notably, residues within the binding pocket displayed reduced RMSF values, suggesting that DG binding restricted local conformational flexibility. MM-PBSA binding energy decomposition further revealed that the total binding free energy was primarily contributed by van der Waals interactions (ΔE_vdw) and electrostatic interactions (ΔE_ele), consistent with the molecular docking results, which demonstrated that DG was stabilized in the EGFR binding pocket via hydrophobic interactions and hydrogen bonding (Figure 5F). In summary, the MD simulation results support the molecular docking predictions, confirming that DG binds EGFR with favorable stability and affinity. The combined contribution of van der Waals forces and hydrogen bonding underpins the stable interaction, providing a mechanistic basis for DG’s regulatory potential on the EGFR signaling pathway.

## Discussion

ARDS is a severe clinical condition characterized by disruption of the alveolar-capillary barrier, uncontrolled inflammatory responses, and excessive oxidative stress (17). It remains a major unsolved challenge in critical care medicine due to the lack of effective targeted pharmacological interventions, posing a significant threat to patient survival. Although numerous therapeutic strategies targeting inflammatory mediators, neutrophil infiltration, and oxidative injury have been investigated in recent years, most candidate agents have failed to reach routine clinical use owing to limited efficacy or unacceptable side effects (18, 19). Therefore, the development of novel ARDS therapies with well-defined mechanisms of action and favorable safety profiles remains of substantial theoretical and practical importance.

Natural products, with their multi-target regulatory properties and relatively low toxicity, have attracted increasing attention as promising therapeutic agents (20). DG, a water-soluble derivative of glycyrrhizic acid extracted from *Glycyrrhiza uralensis*, has demonstrated potent anti-inflammatory, antioxidant, and tissue-protective effects in various inflammatory diseases (21, 22). However, to date, no study has systematically evaluated the therapeutic potential and molecular mechanisms of DG in the context of ARDS. In this study, we comprehensively assessed the anti-inflammatory, antioxidant, and histoprotective effects of DG in ARDS through a combination of *in vitro* and *in vivo* models, network pharmacology analysis, molecular docking, and molecular dynamics simulations. Our findings revealed that DG significantly attenuated LPS-induced alveolar epithelial injury, systemic inflammation, oxidative stress, and histopathological damage in a murine ARDS model. These results provided compelling evidence that DG exerted multi-target, multi-pathway regulatory effects on key pathological processes in ARDS, supporting its potential as a promising natural compound for further development as a therapeutic agent for ARDS.

In terms of inflammatory mediator modulation, DG significantly downregulated the expression of classic pro-inflammatory cytokines such as TNF-*α* and IL-6, while upregulating the anti-inflammatory cytokine IL-10 in both in vitro and in vivo models, indicating robust immunomodulatory capacity. These findings are consistent with previous reports of DG’s anti-inflammatory effects in models of colitis and cutaneous injury (23). Notably, this study represents the first systematic demonstration of DG’s anti-inflammatory potential in an ARDS animal model, thus filling a critical gap regarding its application in pulmonary inflammatory disorders. Importantly, in ARDS, the cytokine storm plays a central role in promoting increased capillary permeability, alveolar edema, and the development of diffuse alveolar damage (DAD) (24). The effective suppression of this inflammatory cascade by DG highlights its ability to intervene in key pathogenic events underlying ARDS. Moreover, DG exhibited significant antioxidant effects. It restored SOD activity, reduced levels of MPO and MDA, and markedly inhibited LPS-induced intracellular ROS accumulation at the cellular level. Oxidative stress is a crucial driver of ARDS progression, exacerbating inflammation via NF-κB pathway activation and inducing apoptosis (25). Previous studies have shown that antioxidants such as N-acetylcysteine (NAC) can confer partial protection in ARDS, though their clinical efficacy remains limited (26, 27). In contrast, the broad-spectrum antioxidant effects of DG observed in this study suggest that it may exert upstream regulatory control over oxidative injury through multi-target mechanisms, potentially offering superior efficacy compared to conventional single-target antioxidants. Compared with traditional ARDS candidate drugs, DG holds unique advantages including its natural origin, favorable safety profile, and multi-pathway synergistic effects. While immunosuppressants such as IL-6 receptor antagonists (e.g., Tocilizumab) and corticosteroids (e.g., Dexamethasone) have shown benefits in selected ARDS subtypes, their use is often limited by increased infection risk and interindividual variability in treatment response (28, 29). In contrast, DG, as a natural derivative with broad anti-inflammatory and antioxidant activity, has demonstrated efficacy in a wide range of non-pulmonary inflammatory models with proven safety, supporting its potential application in ARDS. Collectively, this study provides clear evidence of DG’s protective effects against alveolar epithelial injury and systemic inflammation in an LPS-induced ARDS mouse model, thereby offering important preclinical data to support further translational research. However, DG was administered prior to LPS challenge, following dosing strategies commonly used in experimental models of acute lung injury to evaluate protective and mechanistic effects. While this prophylactic design facilitates mechanistic exploration, it does not fully recapitulate clinical ARDS treatment, which is initiated after disease onset. Therefore, future studies should investigate the therapeutic efficacy of DG when administered at post-challenge time points to better assess its translational potential.

Network pharmacology and molecular docking analyses further elucidated the multi-target synergistic mechanisms by which DG exerts its therapeutic effects. At the target level, DG was significantly enriched in key signaling axes closely associated with ARDS pathogenesis, including EGFR, MAPK1, AKT1, and FGFR1, suggesting its potential to simultaneously modulate inflammatory responses, oxidative stress, and cell survival pathways. Previous studies have demonstrated that EGFR plays a pivotal role in regulating alveolar epithelial repair and vascular permeability, while its hyperactivation exacerbates lung injury. MAPK and PI3K-AKT signaling pathways are also known to control inflammatory cytokine release and apoptosis, respectively (30, 31). In this context, DG exhibited high binding affinity to EGFR and other core targets (binding energy < −10 kcal/mol), forming stable interactions through hydrogen bonding and hydrophobic contacts. These results reveal a plausible molecular mechanism by which DG regulates these signaling cascades. MD simulations further supported this hypothesis, showing that the DG–EGFR complex remained highly stable throughout a 100 ns simulation. Indicators such as RMSD, number of hydrogen bonds, and SASA fluctuated within narrow ranges, while binding free energy calculations indicated that van der Waals and electrostatic interactions were the primary contributors to complex stability. These findings suggest that DG can stably occupy the EGFR active pocket and may suppress its downstream signaling. Notably, while certain small-molecule EGFR inhibitors (e.g., erlotinib) have shown some efficacy in ARDS, their clinical use is limited by adverse effects and the development of resistance (32). In contrast, DG, as a natural compound, may exert a more balanced modulatory effect rather than strong inhibition, thereby preserving essential cellular functions while achieving anti-inflammatory and reparative outcomes. The identification of EGFR as a potential target of DG is supported by integrated computational analyses together with consistent anti-inflammatory and antioxidant phenotypes observed in cellular and animal models. However, direct experimental validation of DG–EGFR binding and downstream signaling regulation was not performed. Therefore, EGFR involvement should be interpreted as mechanistically plausible, and further biological studies will be needed to fully elucidate this pathway. Additionally, this study uncovered potential roles of DG in less commonly targeted ARDS pathways, such as lipid metabolism regulation, steroid hormone response, and membrane-associated signaling processes. For instance, GO enrichment analysis highlighted significant associations with “response to lipid/hormone stimulus,” implying that DG may influence metabolic reprogramming in ARDS. These pathological mechanisms are often overlooked and inadequately addressed by conventional therapies. Therefore, the multi-pathway regulatory properties of DG may offer a more comprehensive pharmacological strategy for managing ARDS, a syndrome driven by complex and multifactorial pathogenesis.

This study highlights a multi-scale integrative analytical strategy, systematically combining cellular, animal, network pharmacology, structural biology, and molecular dynamics data to establish a closed-loop framework from pharmacodynamic validation to mechanistic elucidation. Unlike conventional studies that focus solely on efficacy, our research further delineates the interaction logic between DG and key signaling pathways, validating its mechanism of action on core ARDS targets. These findings provide a solid theoretical foundation for future precision drug delivery, target selection, and clinical translation. Although this study primarily centers on EGFR and its associated signaling networks, the extensive target coverage of DG observed in the PPI network suggests that future investigations are warranted to explore its long-term effects on alveolar barrier repair, immunometabolic regulation, and pulmonary interstitial fibrosis. Nonetheless, certain limitations should be acknowledged. First, pharmacokinetic profiling and pulmonary tissue distribution of DG were not evaluated. Despite its favorable water solubility and tissue affinity, the lung-targeting efficiency and optimal administration route of DG in ARDS remain to be clarified. Second, long-term outcomes and pulmonary function recovery were not assessed in this study, necessitating the inclusion of chronic-phase ARDS models for further validation. Moreover, ARDS is increasingly recognized as a heterogeneous syndrome comprising distinct biological subphenotypes, including hyper-inflammatory and hypo-inflammatory forms. The LPS-induced model used in this study primarily represents a hyper-inflammatory phenotype, characterized by excessive cytokine release and oxidative stress. Accordingly, the protective effects of DG observed herein are most relevant to inflammation-dominant ARDS. Whether DG exerts comparable benefits in hypo-inflammatory or other ARDS subphenotypes remains to be determined and warrants further investigation using phenotype-stratified models or biomarker-guided approaches. In summary, this study is the first to systematically demonstrate the multifaceted protective effects of dipotassium glycyrrhizinate in ARDS. By alleviating lung injury and promoting repair through anti-inflammatory, antioxidant, and EGFR-targeted mechanisms, DG presents both theoretical innovation and promising translational potential. Future investigations focused on dosage optimization, delivery strategies, combination regimens, and long-term safety assessments will provide a robust foundation for the development of DG as a novel natural therapeutic agent for ARDS.

## Conclusion

DG effectively suppressed inflammatory response, mitigated oxidative stress, and improved lung histopathology in both cellular and animal models. And DG targeted key proteins such as MAPK1, EGFR, and FGFR1, modulating PI3K-AKT, EGFR, and HIF-1 pathways involved in ARDS pathogenesis. Molecular docking and dynamics simulations further confirmed its stable binding to EGFR target, supporting a regulatory mechanism. Overall, DG shows promise as a novel ARDS intervention candidate.

## Materials and methods

### Chemicals and reagents

Dipotassium glycyrrhizinate (DG; purity ≥ 98%) was purchased from MedChemExpress (MCE, China). Lipopolysaccharide (LPS, *Escherichia coli* O111: B4) was obtained from InvivoGen (USA). Dulbecco’s Modified Eagle Medium (DMEM), fetal bovine serum (FBS), and penicillin–streptomycin (P/S) were purchased from Gibco (USA). The reactive oxygen species (ROS) probe DCFH-DA was purchased from Beyotime Biotechnology (China). Enzyme-linked immunosorbent assay (ELISA) kits for IL-6, TNF-*α*, and IL-10 were obtained from Multi Sciences (China). Other reagents were of analytical grade. Assay kits for myeloperoxidase (MPO, A044-1-1), superoxide dismutase (SOD, A001-3-2), and malondialdehyde (MDA, A003-1-2) were purchased from Nanjing Jiancheng Bioengineering Institute (China).

### Cell viability assay

The human alveolar epithelial cell line A549 (ATCC CCL-185) was cultured in DMEM medium supplemented with 10% FBS and 1% penicillin–streptomycin at 37 °C in a humidified incubator with 5% CO₂. Cell viability was evaluated using the Cell Counting Kit-8 (CCK-8, Beyotime). A549 cells in logarithmic growth phase were seeded into 96-well plates at a density of 5 × 10^3^ cells/well, with three technical replicates per group. After adherence (~24 h), cells were treated with various concentrations of DG (0, 31.25, 62.5, 125, 250, 500, 1000 μg/mL) in serum-free DMEM to avoid serum interference. The final volume per well was adjusted to 100 μL. Control wells received the same volume of vehicle (drug-free medium). After 24 h of incubation, 10 μL of CCK-8 solution was added to each well, and plates were incubated for an additional 2 h. The absorbance at 450 nm was measured using a microplate reader (33). Each experiment was repeated three times, and the relative cell viability (compared to the control group) was calculated and statistically analyzed to evaluate the cytotoxicity of DG on A549 cells.

### Intracellular ROS detection

Human alveolar epithelial A549 cells were cultured in high-glucose DMEM supplemented with 10% FBS and 1% penicillin–streptomycin at 37 °C in a humidified atmosphere containing 5% CO₂. For ROS detection, cells were seeded into 6-well plates and allowed to reach 70–80% confluence. Cells were first stimulated with lipopolysaccharide (LPS, 10 μg/mL) for 2 h to induce an inflammatory response, followed by treatment with DG at different concentrations (125, 250, and 500 μg/mL) for an additional 24 h. After treatment, cells were incubated with 10 μM DCFH-DA at 37 °C for 30 min in the dark, then washed three times with PBS. Fluorescence intensity was measured using a multifunctional microplate reader with excitation at 488 nm and emission at 525 nm (34). The level of intracellular ROS accumulation was compared across treatment groups. All experiments were performed with at least three independent biological replicates.

### Quantitative real-time PCR for inflammatory cytokines

Following treatment, total RNA was extracted using TRIzol reagent (Invitrogen, USA) according to the manufacturer’s instructions. RNA concentration and purity were assessed by measuring the A260/A280 ratio. One microgram of total RNA was reverse-transcribed into cDNA using the PrimeScript RT Reagent Kit (Takara, Japan).

Quantitative real-time PCR (qRT-PCR) was performed using SYBR Green PCR Master Mix on a QuantStudio 5 real-time PCR system (Thermo Fisher Scientific). Each 20 μL reaction mixture contained 10 μL 2 × SYBR Green Mix, 0.4 μL of each primer (10 μM), 1 μL cDNA template, and 8.2 μL nuclease-free water. The amplification protocol was as follows: initial denaturation at 95 °C for 30 s, followed by 40 cycles of denaturation at 95 °C for 5 s and annealing/extension at 60 °C for 30 s. Melting curve analysis was performed to verify the specificity of amplification (35). Target genes included TNF-*α*, IL-6, and IL-10, with *β*-actin used as the internal reference gene. All samples were analyzed in triplicate. Relative gene expression levels were calculated using the 2^−ΔΔCt method and normalized to the control group. The primer sequences were shown in Table 1.

**Table 1:** Primer sequences used in real-time PCR.

Gene	Forward primer (5′ to 3′)	Reverse primer (5′ to 3′)	Length (bp)
IL-10	GACTTTAAGGGTTACCTGGGTTG	TCACATGCGCCTTGATGTCTG	120
IL-6	ACTCACCTCTTCAGAACGAATTG	CCATCTTTGGAAGGTTCAGGTTG	150
TNF-α	CCTCTCTCTAATCAGCCCTCTG	GAGGACCTGGGAGTAGATGAG	154
β-actin	CACCATTGGCAATGAGCGGTTC	AGGTCTTTGCGGATGTCCACGT	139

### Animals and experimental design

Specific pathogen-free (SPF) female BALB/c mice (6–8 weeks old, 18–22 g) were purchased from Hangzhou Ziyuan Laboratory Animal Technology Co., Ltd. (License No. SCXK (Zhe) 2019–0004). BALB/c mice were chosen because they were used in LPS-induced acute lung injury models and show a reproducible inflammatory response, making them suitable for evaluating anti-inflammatory interventions. Mice were housed in a clean-grade animal facility under controlled conditions (22 ± 2 °C, 40–60% relative humidity, 12-h light/dark cycle) with free access to food and water. After a 7-day acclimatization period, experiments were initiated. All procedures were approved by the Laboratory Animal Ethics Committee of Jiaxing First Hospital (Approval No.: JXYY2022-006), and conducted in accordance with relevant ethical guidelines for animal welfare and protection. Mice were randomly assigned into three groups (*n* = 5 per group): control, LPS, and DG treatment groups. Acute lung injury was induced in the LPS and DG groups by intratracheal instillation of LPS (10 mg/kg, dissolved in PBS) (36, 37). This dose was selected based on previously published studies demonstrating that intranasal or airway delivery of LPS at 10 mg/kg reliably induces acute lung injury and inflammatory responses characteristic of ARDS. Sixteen hours post-induction, mice were anesthetized with sodium pentobarbital (40 mg/kg, intraperitoneal), and blood and lung tissues were collected for further analysis (36). In the DG group, mice received oral administration of dipotassium glycyrrhizinate (DG, 10 mg/kg, dissolved in 0.9% saline) 1 h prior to LPS challenge. Control mice received an equal volume of saline via oral gavage.

### Serum inflammatory cytokine assay

Whole blood was collected and allowed to clot at room temperature. Serum was separated by centrifugation at 3000 rpm for 10 min and stored at −80 °C until analysis. Serum levels of IL-6, TNF-*α*, and IL-10 were quantified using commercially available ELISA kits (Multi Sciences, China) according to the manufacturer’s instructions. Absorbance was measured at 450 nm using a microplate reader (Thermo Fisher Scientific, USA) (38, 39).

### Assessment of oxidative stress markers in lung tissue

After sacrifice, lungs were flushed with PBS to remove blood. The right lung was minced and homogenized in ice-cold saline (1:9, w/v) to prepare 10% tissue homogenates, followed by centrifugation at 12,000 rpm for 10 min. Supernatants were collected for biochemical assays. Commercial kits were used to assess SOD activity, MPO activity, and MDA content. SOD activity was determined based on the xanthine oxidase inhibition method, MPO activity via its catalytic oxidation of o-dianisidine, and MDA levels were measured using the thiobarbituric acid (TBA) method. Results were normalized to total protein concentration and expressed as U/mg protein or nmol/mg protein (40).

### Histopathological analysis of lung tissue

The lower lobe of the left lung was fixed in 4% paraformaldehyde for 48 h, followed by routine dehydration through graded ethanol, paraffin embedding, and sectioning (4 μm thickness). Sections were stained with hematoxylin and eosin (H&E) and observed under a light microscope (Olympus BX53, Japan) (41). Representative images were captured to assess pathological changes, including alveolar structure, interstitial edema, capillary congestion, and inflammatory cell infiltration.

### Network pharmacology analysis

The Traditional Chinese Medicine Systems Pharmacology Database (TCMSP)^1^ was queried using the keyword “Glycyrrhiza uralensis” to obtain active compounds associated with DG. Compounds meeting the criteria of oral bioavailability (OB) ≥ 30% and drug-likeness (DL) ≥ 0.18 were selected as candidate bioactive ingredients. Corresponding known or predicted targets of these compounds were also retrieved from the TCMSP database. Subsequently, disease-related genes associated with ARDS were obtained from the GeneCards database^2^ using the keyword “acute respiratory distress syndrome.” Only protein-coding genes with a relevance score greater than 2 were retained to ensure biological significance and specificity. The intersection between the DG-related targets and ARDS-related genes was then computed to identify potential therapeutic targets of DG against ARDS. These overlapping targets were further imported into the STRING database^3^ with the species limited to “*Homo sapiens*” and a minimum required interaction score set at 0.4 (medium confidence) to construct a protein–protein interaction (PPI) network. The resulting interaction data were visualized and analyzed using Cytoscape software (version 3.10.2), and the NetworkAnalyzer plugin was employed to calculate topological parameters. Core targets were identified based on degree centrality values. Furthermore, GO enrichment analysis—including BP, CC, and MF—and KEGG pathway enrichment analysis were performed on the intersecting targets using the Metascape database,^4^ with statistical significance set at *p* < 0.05 (42, 43). All enrichment results were ranked according to the false discovery rate (FDR)-adjusted *p*-values. Visual representations, including bubble and bar plots, were generated using the Bioinformatics online platform^5^ to construct the compound–target–pathway network and illustrate the potential mechanisms by which DG may exert therapeutic effects in ARDS.

### Molecular docking and molecular dynamics simulation

The two-dimensional structure of DG was obtained from the PubChem database, and its three-dimensional structure was optimized using ChemBio3D. The crystal structures of target proteins were retrieved from the RCSB Protein Data Bank (e.g., EGFR: PDB ID 8A27). Molecular docking was performed using AutoDock Vina to calculate binding affinities, and PyMOL was employed to visualize binding sites and interaction modes. The DG–EGFR complex with the lowest binding energy was selected for subsequent molecular dynamics (MD) simulation.

All MD simulations were conducted using GROMACS 2023. The protein was parameterized using the CHARMM36 force field, while the ligand topology was generated based on the GAFF2 force field (44). Periodic boundary conditions were applied, and the protein–ligand complex was placed in a cubic simulation box solvated with TIP3P water molecules (45). Electrostatic interactions were treated using the particle mesh Ewald (PME) method, and the Verlet cutoff scheme was applied. The system was first subjected to energy minimization, followed by equilibration under the canonical (NVT) ensemble and the isothermal–isobaric (NPT) ensemble, each performed for 100 ps with a coupling constant of 0.1 ps. A cutoff distance of 1.0 nm was used for both van der Waals and Coulomb interactions. Finally, a 100 ns production MD simulation was carried out at a constant temperature of 300 K and a pressure of 1 bar using GROMACS 2023.

### Statistical analysis

All experimental data are presented as mean ± standard deviation (SD). One-way analysis of variance (ANOVA) was used for comparisons among multiple groups to assess pairwise differences. A *p*-value of less than 0.05 was considered statistically significant. All statistical analyses and graphical representations were performed using GraphPad Prism 8.0 software.

## Data Availability

The original contributions presented in the study are included in the article/Supplementary material, further inquiries can be directed to the corresponding author.

## Footnotes

1
https://old.tcmsp-e.com/tcmsp.php

2
https://www.genecards.org/

3
https://cn.string-db.org/

4
http://metascape.org

5
https://www.bioinformatics.com.cn/
